# Study protocol: rationale and design of the community-based prospective cohort study of kidney function and diabetes in rural New Mexico, the COMPASS study

**DOI:** 10.1186/s12882-018-0842-4

**Published:** 2018-02-27

**Authors:** Antonin Jaros, Hafiz A. Sroya, Venita K. Wolfe, Vikas Ghai, Maria-Eleni Roumelioti, Kamran Shaffi, Kai Wang, Vernon Shane Pankratz, Mark L. Unruh, Christos Argyropoulos

**Affiliations:** 1 Department of Internal Medicine, Division of Nephrology, University of New Mexico Health Sciences Center, Albuquerque, NM USA; 2 Department of Internal Medicine, University of New Mexico Health Sciences Center, Albuquerque, NM USA; 3 Community Engagement and Research Core, Clinical and Translational Science Center, University of New Mexico, Albuquerque, NM USA; 4 Institute for Systems Biology, Seattle, WA USA; 5 Section of Nephrology, New Mexico Veterans Hospital, Alburquerque, NM USA

**Keywords:** Chronic kidney disease, Rural, Screening, End-stage renal disease, Biomarkers, Qualitative research, MicroRNAs, Exosomes

## Abstract

**Background:**

Rural areas in the state of New Mexico have been the “ground-zero” for the epidemic of diabetic Chronic Kidney Disease (CKD) in the United States. However, there is limited research about risk factors of diabetic CKD in this area and scarce data regarding the performance of emerging markers of renal filtration and epigenetic biomarkers of renal function and diabetes in this area with its unique ethnic/racial population. We designed the **COMPASS** study as a community-based program in rural New Mexico aiming to screen for CKD and to discover CKD-related translational biomarkers.

**Methods/design:**

The study involves a prospective, longitudinal cohort design involving individuals living in rural New Mexico. Participants undergo a screening for kidney disease using markers of abnormal renal filtration (impaired glomerular filtration rate) or damage (albuminuria). Those found to have CKD on the basis of these tests or those at risk for CKD are enrolled in a prospective longitudinal cohort. We measure markers of renal function, insulin resistance and epigenetics (microRNAs) on patients. Individuals are invited to participate in interviews and focus groups in order to characterize their attitudes towards research and barriers or facilitators to participation in future research studies about kidney disease.

**Discussion:**

This study will provide important data about the local epidemiology of kidney disease in a high-risk rural setting and the utility of emerging renal filtration markers (Beta 2 Microglobulin and Cystatin C), while generating data and methods for the analyses of microRNA biomarkers. The qualitative research subproject will identify factors associated with increased willingness to participate in future translational research projects. With its geographical focus, this study will address a critical disparity in kidney disease research, while generating novel epigenetic data that are relevant for future studies in the general population.

## Background

Chronic kidney disease (CKD) is a frequently unrecognized health problem until end stage renal disease (ESRD) develops and renal replacement therapy (RRT) becomes necessary. The distribution of CKD in the United States (US) assumes a disproportionate importance in rural areas, because of unique socioeconomic factors and limited access to both primary and specialist care [1, 2]. Disparities and barriers to care imply that early CKD diagnosis and therapeutic intervention, which have been shown to slow down the rate of CKD progression and to delay the onset of ESRD, are difficult to implement in rural areas. When ESRD develops, patients residing in rural areas have to travel long distances to receive dialysis services [2]. Screening for CKD, which is not generally recommended in the absence of diabetes and cardiovascular disease [3], may be thus particularly relevant in rural populations.

**Fig. 1:**
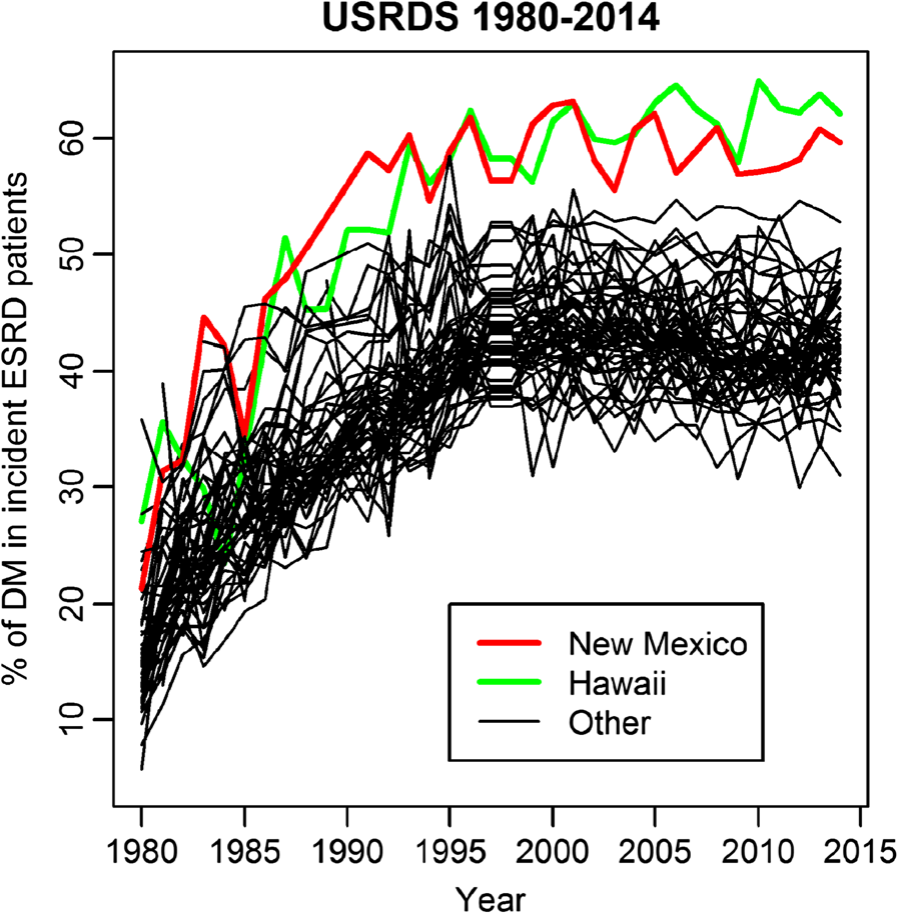
Proportion of incident cases of End Stage Renal Disease attributed to diabetes, United States 1980–2015. The data for this Figure came from querying the public files of the United States Renal Data System through the RenDER web-interface (https://www.usrds.org/render/xrender_home.asp)

The frontier state of New Mexico (NM), has one of the highest prevalence of diabetes as a cause of ESRD in the US. NM is a majority minority state, with only 41% of the population identifying as Non-Hispanic White. A considerable proportion of the population in the state consists of Native Americans and Hispanic Americans residing in rural areas with income levels far below the federal poverty level. Similar to other contexts, the unique ethnic and racial background of the population and the rurality of the state has limited the participation of these patients in clinical epidemiological studies of CKD. Furthermore, there has been virtually no opportunity for these patients to participate in translational research studies evaluating novel biomarkers of renal function and renal damage. This obvious lack of equity in clinical research representation may be a significantly missed opportunity to understand the drivers of diabetic CKD in the general population; for all intents and purposes the state of NM with its rural areas and the state of Hawaii have been “ground zero” for the epidemic of diabetic CKD in the US (Fig. 1).

It is imperative to develop a new approach to simultaneously meet the needs for CKD screening in rural populations and translational research programs that enhance our understanding of CKD, while addressing research disparities in this geographical area. The ***Com****munity Based Study of the E****p****idemiology of Chronic Kidney Dise****as****e in Cuba New Mexico and* ***S****urrounding Areas (****COMPASS****)* project was designed as a community-based kidney health screening and translational biomarker research program for participants in a geographically defined area in rural NM. The primary objective of this manuscript is to describe the design and implementation of a dual use, community-based CKD screening and translational research program and to provide preliminary data about the baseline characteristics of the screened cohort during the first 18 months of the project.

## Methods/design

### Setting

In this study we utilize quantitative, translational biomarker, and qualitative methods for data collection. The study is designed as a prospective, longitudinal cohort involving participants who are not undergoing dialysis and who have not received a functioning kidney transplant. Individuals with these characteristics will be invited to participate in a kidney health screening event. Patients with negative screening for CKD and risk factors for CKD will be offered the opportunity to be rescreened at yearly intervals. Standardized letters are used to communicate screening results and incidental laboratory findings to study participants. The overall duration of the project is anticipated to be 5 years. Clinical laboratory measurements and microRNA discovery activities are handled by the Clinical and translational Science Center (CTSC) of the University of New Mexico (UNM) and the Institute for Systems Biology in Seattle, respectively.

### Inclusion criteria

Patients who live in/around Cuba, NM (within 20 miles) and are older than 18 are allowed to participate in this study. Cuba is a small village (population of 735 according to the 2010 census, all classified as rural); it is a regional hub which provides essential government functions for the rural areas lying between the cities of Rio Rancho in the South and Farmington in the North along the major highway US - 550. It is one of the most diverse residential areas in NM and in the US (diversity index of 85/100 versus national average of 60/100) with 36.5% of families and 41.3% of the population below the poverty line. There is a high prevalence of diabetic ESRD in the state of NM and along the communities around US-550; the high prevalence and the hub role of the village for a very large geographic area provided the justification for selecting this community as a study site. Cuba fosters a dialysis unit that provides renal support to patients residing in a 50 mile radius area around the village. This dialysis unit is operated by the Dialysis Clinic Inc., a nonprofit medical corporation; the unit also houses a CKD clinic operated by the UNM. The dialysis unit with its in-house lab (to process samples for the routine monitoring of the dialysis patients), clinical equipment and space makes itself a natural choice for a research site. Other inclusion criteria were: age range 18–80 years, ability to provide informed signed consent and having a residential address in or around Cuba, NM (within 20 miles) or originating from Cuba but living in Albuquerque, NM.

### Exclusion criteria

The only two exclusion criteria are being on renal replacement therapy (hemodialysis, or peritoneal dialysis) or having received a functioning kidney transplant.

### Research objectives

The primary *end point* of this study is the prevalence of participants with CKD G3–5 (defined as estimated glomerular filtration rate, eGFR < 60 ml/min/1.73m^2^ based on the CKD-EPI equation) and CKD A2–3 (persistent albuminuria) at each visit. The study has a number of *secondary, biomarker oriented end points* which are geared towards understanding the relationship of emerging markers of renal filtration, e.g. Beta 2 Microglobulin (β2M) or Cystatin C (CysC) to levels of inflammation, indices of insulin resistance and other laboratory tests of renal function (Table 1).

**Table 1:** Secondary Biomarker End Points

Concentration of β2M versus eGFR and markers of inflammation (high sensitivity C-reactive protein or hsCRP) in all evaluable participants
Laboratory markers of glycemic control (HgbA1C), and insulin sensitivity (HOMeostatic model Assessment (HOMA) for assessing beta-cell function and Insulin Resistance or HOMA-IR, C-peptide index) versus eGFR in all screened participants
Relation between emerging renal filtration markers (β2M and CysC) in participants with CKD
Relation of β2M, CysC and insulin sensitivity at each time point to rate of decline of the eGFR (ΔeGFR)
Relation between inflammation (hsCRP), eGFR and ΔeGFR
Relation of laboratory markers of CKD (anemia, calcium, phosphorus, parathyroid hormone, vitamin D), abnormal serum electrolytes, osmoregulation and urinary biomarkers (hematuria, albuminuria, proteinuria, urine electrolytes) to eGFR and ΔeGFR
Relation of progressive CKD (ΔeGFR > 3 ml/min/1.73m^2^ per year) status in the community to personal, medical and family history risk factors

Qualitative research methodologies are applied to understand the participant’s perceptions and attitudes towards research activities in the area of CKD [4, 5]. The analysis of these factors is a secondary, non-biomarker oriented aim of our study. The study has a single major *exploratory* outcome, the description of relationships between clinical laboratory parameters of renal function and damage (e.g. albuminuria) to circulating plasma or urinary microRNAs. MicroRNAs are an emerging class of rational, biologically plausible biomarkers of organ damage in kidney disease. This research objective was added to the study protocol after receiving an overwhelming feedback from our first fifty participants that this study provided value to the community.

### Study procedures

#### Recruitment

The study is advertised using flyers (e.g. the health center, the library, the police station, the community center) and whole page advertisements in the local newspaper. A community engaged specialist distributes these flyers and informs village residents about the study. We also accept referrals through the community, e.g. from other participants.

#### Patient’s flow

All participants undergo a laboratory evaluation of blood and urine in addition to the collection of medical history items and physical exam. Patients are classified as having a positive CKD screen on the basis of impaired eGFR (CKD G3–5) or albuminuria (CKD A2–3) in this initial testing. Patients with a positive CKD screen are given the option to be followed up longitudinally with health questionnaires and extended laboratory examination after 1 year (Visit 2, Month 12). Patients without evidence of CKD will generally not be rescreened unless they are at high risk of CKD on the basis of clinical risk factors for the development of CKD: personal or family history of hypertension or diabetes or cardiovascular disease, family history of CKD or ESRD, known history of hepatitis B/C, rheumatologic or autoimmune disease, age older than 50 years.

#### Medical history and physical examination

After providing informed signed consent (Visit 1, Month 0) all participants fill out a medical questionnaire and undergo a targeted physical examination. The medical questionnaire incorporates elements from previous community screening efforts for CKD (e.g. National Kidney Foundation, Kidney disease Early Evaluation Program, KEEP [6, 7]) and national health surveys (NHANES) [8, 9]. Demographical information (age, gender, race, ethnicity), social history, family history, list of medications, attained education level are also collected. Physical examination consists of measurement of systolic and diastolic blood pressure in both arms using appropriately sized cuffs, recording of height and weight for BMI calculation, number of respirations and blood oxygen saturation.

#### Clinical laboratory measurements

All participants undergo a *screening* laboratory evaluation modelled after the KEEP screening program [10], that was based on measurement of renal filtration impairment and albuminuria [11], but including an extensive index of glucose metabolism and insulin resistance. An extensive laboratory testing is performed on the plasma and urine samples with a positive CKD screen (Table 2). The purpose of this extensive testing is to characterize the endocrine and tubular functional adaptation of the kidneys in patients with evidence of renal filtration impairment (decreased eGFR) or kidney damage (albuminuria).

**Table 2:** Clinical Laboratory Determinations

Screening tests	Additional tests in participants with CKD
Blood/Serum	
Complete Blood Cell Count (including Hemoglobin and Hematocrit)	Magnesium
Hemoglobin A1C	Lead
C-peptide	Parathyroid Hormone
Insulin	Vitamin D, 25-Hydroxy
Full non-fasting lipid panel	
Renal Function Panel: sodium, potassium, chloride, carbon dioxide, glucose (random/non-fasting), blood urea nitrogen, creatinine, albumin, phosphorus	
Uric Acid	
High Sensitivity C-reactive Protein	
Cystatin C	
Βeta 2 Microglobulin	
Urine	
Automated Urinalysis	Urine Urea Nitrogen
Urine Creatinine	Urine Electrolytes: Sodium, Potassium, Chloride, Calcium, Phosphorus, and Magnesium
Urine Albumin and Total Protein	Urine Uric Acid

Various indices of insulin sensitivity are assessed during the project: a) the C-peptide, an index of insulin release, b) the non-fasting C-peptide to insulin ratio, c) the HOMA-IR [12], and d) the Quantitative Insulin Sensitivity Check Index (QUICKI). The last two indexes are mathematical transformations of the glucose and insulin plasma levels. Measurements of these biomarker values in the *fasting* state provides the input for the HOMA-IR and QUICKI calculations. In this project we use, non-fasting, random sampling for glucose, insulin and C-peptide as it is far more convenient and sufficient for population level studies such as the presented one [13]. CysC is measured with a low cost Luminex research-grade assay (R&D systems), which is cheaper than the standard immunonephelometry assays used by clinical laboratories. CysC is measured with both techniques in a calibration sub-study; the latter will provide linear calibration factors to convert the Luminex assay readings to immunonephelometry ones. A cold chain was set up for the transport of specimens collected in Cuba, NM to the UNM CTSC laboratory. The latter aliquots and stores samples for further testing at the TRICORE clinical chemistry laboratory, the CTSC T1 lab and the Institute for Systems Biology. Establishment and maintenance of the cold chain ensures that samples can be used for the accurate and precise measurements of all analytes by respecting the temperature pre-analytic requirements of all assays utilized.

#### Epigenetic microRNA biomarkers

An exploratory aim of this study is to identify circulating (plasma) and urinary microRNA biomarkers of CKD. Secondarily, we are interested in the relation of microRNAs to markers of insulin resistance and glycemic control. MicroRNAs will be assessed in three different fractions: total biofluid (plasma or urine), Extracellular Vesicles (“exosomes”) [14] and concentrated Extracellular Vesicles-depleted fractions. Assaying these three fractions within each biofluid matrix will generate a large first in-kind dataset about the compartmentalization of microRNA biomarkers that correlate with markers of kidney damage, impaired renal filtration and insulin resistance. Due to the lack of general consensus about microRNA isolation protocols [15], and the critical importance of the latter for microRNA biomarker discovery [16], we provide a detailed description of our protocol.

##### Sample Preparation for microRNA biomarker discovery

For each sample, 600 μl of urine is spun at 2000 g at 4 °C for 30 min to remove cellular debris. The clarified supernatant (~ 500 μl) containing cell-free urine and 500 μl of plasma are used for either direct-RNA isolation, or subsequent isolation of Extracellular Vesicles. For Extracellular Vesicles isolation, 250 μl of biofluid matrix (either plasma or urine) is applied through a size-exclusion column (Izon Science, Cambridge MA) with de-gassed 1X phosphate buffered saline. The first six 500 μl void fractions are discarded, and the subsequent Extracellular Vesicles -containing fractions (7–10) are collected in a single pooled 2 ml volume. The following fractions (11–35) depleted of Extracellular Vesicles are collected in a single 15 mL tube. Both the pooled Extracellular Vesicles and Extracellular Vesicles-depleted fractions are concentrated separately to ~ 100 μl using Amicon 10 K centrifugation filters (EMD Millipore, Billerica MA) spun in a swing-bucket rotor at 4000 x g at 4 °C for 20 min. Hence, a microRNA is isolated from (1) 250 μl of cell-free biofluid, (2) ~ 100 μl of concentrated Extracellular Vesicles fractions and (3) ~ 100 μl of concentrated Extracellular Vesicles-depleted fractions using the miRNeasy Micro kit (QAIGEN, Germantown MD). The RNA is eluted with 14 μl of nuclease-free H_2_0 and quality is assessed using an Agilent 2100 Bio-analyzer (Agilent Technologies, Santa Clara CA) with a RNA (Pico) chip. An in-house small RNA sequencing library construction method that utilizes adapters with 4 degenerated bases to reduce adapter-RNA ligation bias will be used (Etheridge et al. in prep). Briefly, 6 μl (~ 20 ng) of RNA is used as input for the protocol. The 3′ adapter (/5rApp/(N:25252525)(N)(N)(N)TGGAATTCTCGGGTGCCAAGG/3ddC/) is ligated to the RNA first, followed by ligation of the 5′ adapter (/A*C*A*C*GUUCAGAGUUCUACAGUCCGACGAUC(N:26282026)r(N)r(N))r(N)). Ligated RNA is used to make complementary DNA, which is then polymerase chain reaction (PCR) amplified for 5 cycles and cleaned up using Solid Phase Reversible Immobilization (SPRI) beads. Purified PCR product is size-selected for a 127–156 base-pair, bp size range (performed using a Pippin HT automated size-selection instrument (Sage Science, Beverly MA)), then run for an additional 15–18 cycles, purified, and then size selected again. Individual library concentrations are measured using the NEBNext Library Quant Kit for Illumina (New England Biolabs, Ipswich MA) and adjusted to a final pooled concertation of 2 nM and run on NextSeq sequencer (Illumina, San Diego CA). For a detailed overview of the steps involved in the construction of microRNA libraries see the supplementary methods of our short RNA sequencing analysis companion paper [17].

#### Qualitative data collection

According to the principles of grounded theory methodology, qualitative interviews and focus groups will be conducted until saturation, defined as when no new concepts or themes emerge, is reached. Based on these principles, we planned a minimum of 4 focus groups (and up to 10) with 5–8 community participants per group in order to reach saturation of themes relevant to the study’s specific aims. For the qualitative interviews, guidance from qualitative analysis work suggests that 10–20 interviews are adequate for saturation. Due to the high enrollment size for this study, a total of 50 interviews is planned to serve as an adequate sample size for this study. Interview scripts explore community participant’s thoughts around participating in research including reasons why the participant joined research, concerns around research, and concerns around providing blood samples. Participants are asked to discuss their understanding of chronic illnesses pre and post follow up visits. Participants are implored to provide feedback on the current study’s practices and procedures, what the researchers can do to improve in current practices, and what topics are important to explore in future research activities in their community. The Community Engagement Research Core (CERC) will serve as the central repository for the qualitative data from the focus groups and interviews. The focus groups and interviews are audio recorded. These recordings are secured by the study coordinator and submitted for transcription. Once transcribed, the audio recording is saved until the end of the study. The subjects do not have the opportunity to review the recordings. At the completion of the study the audio files will all be deleted.

#### Statistical analyses

The size of the cohort (anticipated enrollment target of 500, 80% of the size of the population living around the study site) was determined foremost by the needs for a clinical screening program for CKD in the target population. A formal power calculation shows that this is the size required to estimate the prevalence rate with a precision of 3%, assuming a national background prevalence rate of CKD equal to 14% [18].

Descriptive methods (summary statistics: means and standard deviations, medians/interquartile range, graphical: histograms/kernel density estimators) will be used to summarize and visualize the measurements obtained in this study. Relationships between variables will be explored via means of parametric generalized linear regressions and flexible generalized additive models based on thin plate regression splines. These models allow one to undertake a non-linear modeling between outcome (e.g. CKD status) and exposure (e.g. β2M concentration) in a data-driven fashion [19]. It is likely that certain outcome variables (e.g. CKD status or the level of eGFR), will exhibit additional variation than the one anticipated by conventional parametric models (e.g. logistic regression). In such a case, we will apply the approach of Generalized Linear Regression for Location, Scale and Shape (GAMLSS) to analyze study findings. GAMLSS [20, 21] allow one to simultaneously model the variance (scale) and variance (shape) parameters of statistical distributions so as to capture the heteroscedastic, non-linear form of many real world measurements using spline models. Concordance between measures of renal filtration will be assessed with the kappa statistic. The c-statistic will be used to assess the predictive ability of combinations of laboratory tests for progressive CKD.

##### Short RNA sequencing measurements

MicroRNA measurements (counts of sequences of distinct microRNAs) will be analyzed by our recently described Linear Quadratic Normal (LQNO) GAMLSS model [17]. This LQNO model is a statistical regression framework for the analyses of short RNA sequencing counts that takes into consideration the statistical processes underlying this complex measurement. We have shown that the LQNO outperforms six popular algorithms for short RNA sequencing in terms of sensitivity, specificity, false positive and false negative rates. In fact, the LQNO can generate unbiased differential expression ratios that are correctly classified in direction and accurately estimated in magnitude. Hence, they do not need to be verified by the gold – standard technique of PCR. For the purpose of this work, we will use the LQNO to undertake differential analysis between patients with and without CKD, and patients with and without diabetes. The LQNO will also be used to report absolute concentrations of microRNA counts that have been calibrated against synthetic mixes of microRNAs with known composition. This calibration can remove an excess of 40% of the bias in seq counts, clearing the path for advances systems modeling of the complex, multivariate short RNA sequencing measurements.

##### Qualitative data analysis

Computer analysis through the use of NVivo qualitative analysis software (QSR International) will be used to organize the data across multiple transcripts. All files will be imported into the NVivo qualitative analysis software. The de-identified transcripts will be distributed to authorized members of the study team for analysis. Each transcript will be reviewed and coded by the team of researchers who agree, by consensus, on the coding scheme/analytic template (one for the qualitative interviews, and one for the focus groups), which will be based on the themes, categories, and codes that are most prevalent throughout the transcripts. The study team will conduct the analysis in NVivo, and will create an analytic summary based on the findings.

## Discussion

The **COMPASS** study was designed as a community based kidney health screening and translational biomarker discovery program in rural NM. This is an area of extremely high prevalence of CKD and diabetes, which in many aspects can be considered to be a ground-zero for the epidemic of diabetic CKD and ESRD in the US. As a community health screening project [10], **COMPASS** aims to characterize the prevalence and risk factors of diabetes, insulin resistance and CKD in a well-defined, diverse rural area that is located away from large population centers. This setting exemplifies the challenges of providing renal care in the rural US. As a biomarker discovery project, **COMPASS** will deliver a large-scale description of the microRNA profiles in patients with CKD and diabetes, but also normal controls using state of the art protocols for the measurement and analysis of microRNA profiles. Hence, the proposed study protocol will not only provide crucial local epidemiological data, but will also inform the design of larger studies in the area of microRNA biomarker research.

This cohort study combines quantitative, translational biomarker, and qualitative research methods in order to address the unique challenges for renal research and care in this particular setting. Such challenges arise from both health care and research disparities in the target population. The remoteness of the area and the overwhelmed clinical infrastructure for care, imply that many patients who should receive screening for CKD based on recent guidelines may not be provided with the opportunity to do so. At the same time, this unique population of predominantly Native Americans and Hispanic Americans has been largely excluded from existing CKD studies. Systematic reviews have shown that these groups are willing to participate in research if they are given the opportunity to do so [22, 23]. Nevertheless, research on emerging laboratory markers of renal filtration, e.g. β2M and beta-trace protein [24] or CysC [25] are limited to single, cross-sectional assessments on relatively small samples. The **COMPASS** study will nearly double the public record of these measurements in Native Americans, while providing an ecological control of Caucasians and Hispanic Americans for the concentration of these biomarkers in plasma.

There are no data on novel epigenetic microRNA biomarkers of diabetes and CKD in this population. Recent research by our group [26, 27] and others (reviewed in [5, 28]), suggests that microRNAs may be a particularly relevant class of biomarkers for diabetes and CKD. MicroRNAs are small non-coding RNAs that control the expression of other genes at the post-translational level. They have been implicated in the pathogenesis of diabetes, initiation and progression of CKD and cardiovascular dysfunction which is the most common comorbid condition in CKD. Researching microRNA biomarkers in the **COMPASS** population which is at extremely high risk of diabetic CKD, not only addresses an obvious research disparity but may inform future studies about the utility of these biomarkers in the general population. In fact the **COMPASS** project has already generated significant methodological advances in the statistical techniques for the analysis of microRNA data [17]. These techniques not only address the variability [29, 30] and bias [31–33] in the short RNA sequencing measurements, but are able to estimate group differences in expression with high accuracy and precision. When applied to the measurements of microRNAs in different bio fluidic compartments (e.g. Extracellular Vesicles and Extracellular Vesicles -depleted fluids), these techniques may yield markers of renal function and renal damage that have higher sensitivity and specificity than the existing laboratory measurements. Indeed this possibility has been suggested in our previous work in the nephropathy of type 1 diabetes [27].

### Summary

The **COMPASS** study combines a community based kidney health screening with a translational biomarker discovery program. The study will provide much needed data for the local epidemiology of CKD in an area with a high prevalence of diabetes, while generating a wealth of novel epigenetic, microRNA biomarker data and the techniques to analyze them. The results of **COMPASS** may form the basis for subsequent projects in other rural areas with substantial health and research disparities, while informing research on microRNA biomarkers in the general population.

## Abbreviations

CKD: Chronic Kidney Disease
CysC: Cystatin C
eGFR: Estimated Glomerular Filtration Rate
ESRD: End Stage Renal Disease
HOMA-IR: HOMeostatic model Assessment (HOMA) for assessing β-cell function and Insulin Resistance
hsCRP: High sensitivity C-Reactive Protein
PCR: Polymerase Chain Reaction
QUICKI: Quantitative Insulin Sensitivity Check Index
β2M: Beta 2 Microglobulin
ΔeGFR: Rate of decline in the estimated Glomerular Filtration Rate
